# A Comparative Study of Segregation Patterns in Belgium, Denmark, the Netherlands and Sweden: Neighbourhood Concentration and Representation of Non-European Migrants

**DOI:** 10.1007/s10680-018-9481-5

**Published:** 2018-03-21

**Authors:** Eva K. Andersson, Bo Malmberg, Rafael Costa, Bart Sleutjes, Marcin Jan Stonawski, Helga A. G. de Valk

**Affiliations:** 1 Department of Human Geography, Stockholm University, Stockholm, Sweden; 2 Faculty of Economic, Social and Political Sciences and Solvay Business School, Vrije Universiteit Brussel, Brussels, Belgium; 3 Netherlands Interdisciplinary Demographic Institute (NIDI)/KNAW/University of Groningen, the Hague, the Netherlands; 4 Department of Sociology and Human Geography, University of Oslo, Oslo, Norway; 5 Department of Demography, Cracow University of Economics, Kraków, Poland

**Keywords:** Segregation, Comparison, Non-European immigrants, Concentration, Representation, Belgium, Denmark, The Netherlands, Sweden

## Abstract

In this paper, we use geo-coded, individual-level register data on four European countries to compute comparative measures of segregation that are independent of existing geographical sub-divisions. The focus is on non-European migrants, for whom aggregates of egocentric neighbourhoods (with different population counts) are used to assess small-scale, medium-scale, and large-scale segregation patterns. At the smallest scale level, corresponding to neighbourhoods with 200 persons, patterns of over- and under-representation are strikingly similar. At larger-scale levels, Belgium stands out as having relatively strong over- and under-representation. More than 55% of the Belgian population lives in large-scale neighbourhoods with moderate under- or over-representation of non-European migrants. In the other countries, the corresponding figures are between 30 and 40%. Possible explanations for the variation across countries are differences in housing policies and refugee placement policies. Sweden has the largest and Denmark the smallest non-European migrant population, in relative terms. Thus, in both migrant-dense and native-born-dense areas, Swedish neighbourhoods have a higher concentration and Denmark a lower concentration of non-European migrants than the other countries. For large-scale, migrant-dense neighbourhoods, however, levels of concentration are similar in Belgium, the Netherlands, and Sweden. Thus, to the extent that such concentrations contribute to spatial inequalities, these countries are facing similar policy challenges.

**Electronic supplementary material:**

The online version of this article (10.1007/s10680-018-9481-5) contains supplementary material, which is available to authorized users.

## Introduction

The aim of this paper is to compare levels and patterns of non-European migrant segregation in four different countries: the Netherlands, Denmark, Sweden and Belgium. Comparing residential segregation between countries helps scholars to analyse the causes and consequences of segregation, and also helps them to suggest policy measures against segregation. Three questions will be in focus. (1) To what extent are there differences in the concentration and representation of non-EU migrants across various spatial scales? (2) How can differences in segregation patterns across four national contexts be explained by differences in structural factors? (3) To what extent has spatial segregation reached such levels that the social cohesion of different member states is endangered?

Residential segregation implies the relatively strong presence of a specific group in some spatial units combined with a relatively low presence in others (Massey and Denton 1988). In particular, the spatial segregation of non-European migrants is a topic of academic and policy debate in several countries across Europe. However, the levels of segregation and their specific spatial patterns are different across countries, as well as across cities and regions within countries. These differences may be explained by the large variation in the size and composition of the non-European migrant population across countries, as well as by cross-national variety in structural conditions. For example, a recent comparative study identified globalisation, social inequalities, welfare regimes, housing systems and occupational structures as the main structural factors shaping socioeconomic segregation (Tammaru et al. 2016).

Making country comparisons is important since this makes it possible to discuss the influence of national policies on segregation patterns. Although the countries we study are similar in terms of welfare policies, there are large differences in terms of the housing market, placement policies and in the composition of non-European migrants, which may be reflected in different spatial segregation patterns.

Earlier comparative studies on residential segregation usually were carried out on the geographical level of cities or city regions. Examples include the study by Musterd and van Kempen (2009) on ethnic segregation, the recent study by Tammaru et al. (2016) on socioeconomic segregation and the work of Musterd and Ostendorf (1998) and Musterd (2005) on both types of segregation. However, some of the factors that previous studies identified as crucial for shaping spatial segregation-social inequality, namely welfare regimes and housing market policies (Tammaru et al. 2016), play a role at the national scale and are just as likely to influence segregation both within and across non-metropolitan and metropolitan areas. Segregation is a phenomenon that is present wherever there is population and is thereby not limited to large metropolitan areas or big cities. In fact, patterns of segregation are also visible in small towns and cities. Segregation can even exist between rural settlements. Indeed, a number of recent studies have highlighted residential segregation in non-metropolitan areas (Lichter et al. 2012; Östh et al. 2014). It is therefore interesting to compare differences in segregation patterns between different countries while focusing on urban, suburban and rural areas.

One important finding in our study that focuses on conditions in 2011 is that segregation patterns at the lowest geographical scale are remarkably similar, almost identical in fact, across the four countries we study. This is a finding that we have not seen reported before, and it points to a strong need to consider how such similar outcomes can result in different countries. At larger scales, we find that the level of segregation is markedly higher in Belgium compared to Denmark, Sweden, and the Netherlands. This points to the possibility that differences in policy are more important for large-scale patterns than for small-scale patterns of segregation. Our analysis also shows that the proportion of the population that lives in migrant-dense neighbourhoods is higher in countries with a large non-European migrant population. This is not unexpected but underlines that segregation can become a greater challenge in such countries. On the other hand, in Sweden and the Netherlands, relatively high proportions of non-European migrants are also found in neighbourhoods dominated by natives and European-born persons, a pattern suggesting that policies that are in place in these two countries have had some success in preventing segregation. Overall, the results we present show that using methods that ensure comparability and a multiscalar approach opens up possibilities for evaluating competing theories of segregation and assessing the impact of different policies.

## Previous Comparative Studies on Segregation Patterns

Several previous studies made cross-country comparisons of residential segregation. Some studies compared segregation in Europe to that in the USA. The overall conclusion of these studies is that segregation levels in Europe are more modest (Friedrichs et al. 2003; Musterd and Ostendorf 1998) than in the USA. Musterd and Ostendorf (1998) studied segregation in different Western cities, including Stockholm, Amsterdam and Brussels. They concluded that segregation is stronger in Brussels than in Amsterdam and Stockholm, but levels are low compared to the American and South African contexts they covered in their work.

A number of studies compared segregation across metropolitan regions in Europe. Musterd (2005) compared scores on the dissimilarity index (DI) for ethnic minorities between different European cities based on results published from the late 1990s to the early 2000s. His work showed that German cities, as well as Oslo and Vienna, have the lowest segregation levels (DI), followed by Dutch cities with the exception of Rotterdam. Belgian cities generally have higher segregation levels. In a later, similar study, Musterd and van Kempen (2009) again collected DI-scores from European cities for several years and stated that the dissimilarity indices were stable over time, and even decreased somewhat.

In a study of segregation patterns including 16 European countries clustered into different welfare regimes (social-democratic, corporatist, liberal or Latin Rim) until the mid-1990s, Arbaci (2007) concluded that “welfare arrangements are critically important” (p. 429). Generally, cities in corporatist welfare systems have the lowest levels of spatial segregation because of their ‘unitary’ or ‘integrated’ rental systems, and cities in liberal welfare states have the highest degree of segregation due to a ‘dualist’ rental system (Arbaci 2007). In dualist systems, public housing is a restricted sector for low-income households, whereas social housing is competing on even terms with private renting in unitary rental systems (Kemeny 2006; Skifter Andersen et al. 2016).

In the literature, the availability of housing and the opportunities for different ethnic minority groups to gain access to housing has been mentioned as an important driver of ethnic segregation in both the USA (South et al. 2011) and in Europe (Musterd and van Kempen 2009; Skifter Andersen et al. 2016). One reason is that the social rented sector is not equally distributed across urban space (Friedrichs et al. 2003; Tammaru et al. 2016). This translates into ethnic segregation since non-Western migrants are over-represented in lower socioeconomic strata and groups with a low socioeconomic status are over-represented in the social rented sector. It also implies that ethnic segregation will overlap closely with socioeconomic segregation.

Skifter Andersen et al. (2016) compared ethnic segregation in four Nordic capitals (Stockholm, Copenhagen, Helsinki and Oslo) with similar social-democratic welfare state systems but different housing markets and spatial distributions of housing tenures. They found that generally, the degree of ethnic segregation increases with the size of the immigrant population: the strongest segregation was found in Stockholm, with the largest immigrant population. A lack of local mixing of tenure types, however, also influenced the level of segregation. Still, there is no straightforward relationship between the housing system and the level of segregation. Ethnic segregation is stronger in a restricted social sector, but if housing policies guarantee a mixture of tenure in neighbourhoods, the level of segregation for the neighbourhood as a whole may be lower, as the example of Helsinki showed (Skifter Andersen et al. 2016).

Avoidance or flight by natives also influences the degree of segregation. In the European context, some evidence of a higher likelihood for natives to leave migrant-dense neighbourhoods was found for the UK (van Ham and Manley 2009), Denmark and Sweden (Skifter Andersen et al. 2016). Several studies suggest that natives tend to avoid such areas (Bråmå 2006; Zorlu and Latten 2009). Examples of constraints that lead to the concentration of migrant groups in certain neighbourhoods are restrictive housing allocation systems and welfare state mechanisms (Musterd and van Kempen 2009; van Ham and Manley 2009).

These previous studies lead us to expect differences in segregation patterns between Belgium, Denmark, the Netherlands and Sweden essentially depending on how factors such as the housing market, the welfare state and spatial planning are arranged in each national context. Regarding the welfare state system, Denmark, Sweden and the Netherlands fall into a social-democratic welfare cluster, whereas Belgium can be characterised as a corporatist welfare state (Arbaci 2007). This is also reflected in the housing market structure. Belgium has a dualist rental system with a limited social rental sector that accounts for only 6.9% of the total housing stock (Vanneste et al. 2008; de Decker 2008; Kesteloot and Cortie 1998), which is considerably smaller than the number of families eligible for public rented housing (de Decker 2008; Kesteloot and Cortie 1998). As early as 1998, different segregation outcomes between Belgium and Sweden were said to be “in part based on the different nature of the housing system” (van der Wusten and Musterd 1998, p. 240). The same study also concluded that an important public housing component can have “a softening effect on segregation levels” (p. 246).

Although the Netherlands, Sweden and Denmark have unitary rental systems with a large stock of public housing, there are still considerable differences between the four housing markets. In 2011, 44% of the total housing stock in the Netherlands belonged to the public rented sector: subsidised dwellings offered by housing corporations, with rents below approximately 600 euros. In the cities, this proportion has always been higher: in Amsterdam, it was approximately 67% in 2011 (Statistics Netherlands 2011). The Dutch social rented sector is considered attractive because of the large proportion of single-family housing and the often good state of dwellings. Although units are generally allocated to low-income households, tenants cannot be evicted when their income increases. As a result, the tenants are mixed in terms of income (Bolt et al. 2008). In recent years, however, urban policies have sought to reduce the stock of social rented dwellings (Savini et al. 2016). The large and diverse Dutch social rented sector explains why segregation levels in Dutch cities are much more modest compared to cities in the UK (Murie and Musterd 1996), where the social rented sector is smaller and spatially more concentrated. Kesteloot and Cortie (1998) compared ethnic segregation in Amsterdam and Brussels for Turkish and Moroccan migrants and concluded that these groups are more strongly segregated in Brussels. Due to the small social rented sector in Brussels and their generally lower incomes, Turkish and Moroccan migrants in Belgium depend on the private residual rented sector, which is mainly found in a restricted number of working-class neighbourhoods.

Another factor influencing segregation patterns is differing policies that regulate the number of people entering the country, when they can enter, who can enter, where they can settle, and the kind of support offered to immigrants living in the country. Sweden, Denmark and the Netherlands have had policies seeking the dispersal of refugee populations (Andersson 2003; Robinson et al. 2004). Concerning the number of entering non-European migrants, before 2015, Denmark was renowned for its low levels of immigration, whereas Sweden, the Netherlands and Belgium had accepted larger numbers. In earlier studies, Scandinavian countries together with the Netherlands were portrayed as similar concerning the organisation of housing (van der Wusten and Musterd 1998). In terms of welfare systems, Denmark and Sweden are considered as belonging to the social-democratic model, whereas Netherlands and Belgium have been classified as hybrids between the conservative and social-democratic model (Kammer et al. 2012). This welfare state classification, thus, to some extent points to similarities rather than differences between the countries in our study.

## The Role of Spatial Scale for Segregation Measurement

Over the past two decades, an increasing number of studies have addressed how residential segregation relates to spatial scale. van der Wusten and Musterd (1998) concluded that their study on segregation in Western cities cannot be considered truly comparative due to barriers related to data acquisition, differences in spatial units and different definitions of ethnic categories. In relation to the scale-dependent nature of segregation effects, they observed that:

When either income or ethnic status differences are very pronounced, exclusion is supposed to follow. In the case of segregation, opinions are more divided. It depends on the spatial scales involved: the larger the units, the higher the probability of exclusion. A major reason why this seems convincing is the provision of a self-sufficient environment within larger units with no incentives to use urban space at large (van der Wusten and Musterd 1998, p. 241).

Similar conclusions were drawn by Musterd (2005), whose study compared levels of ethnic segregation across metropolitan areas in different European countries and acknowledged both conceptual issues and problems related to the scale of measurement and time points in measurement. The different ways in which statistical units are constituted across different areas is referred to as the Modifiable Areal Unit Problem (MAUP) (Openshaw 1984; Östh et al. 2014). MAUP prevents reliable comparisons of segregation levels and patterns between areas of different sizes, as well as between different countries, while ideally, comparative studies use uniform units of measurement (Musterd 2005).

Thus far, most studies on residential segregation have focused on patterns in metropolitan areas and have used administrative (neighbourhoods) and statistical (census tracts) units for segregation measurement. Krupka (2007) argued that the often found relationship between high segregation levels and large city size is spurious and caused by differences in neighbourhood size between larger cities and smaller towns. Census tracts in metropolitan areas generally consist of a single neighbourhood, while neighbourhoods in smaller towns have fewer inhabitants and must be combined in order to fill a census tract of comparable size. Krupka (2007) measured segregation at different spatial scales and found that using smaller areas of analysis diminished the differences between large cities and small ones.

The plea for multiscalar measurements of segregation has intensified over the past few years. Fowler (2016) recently argued that there is no ‘correct’ scale for measuring segregation: it is continuous across scales and should be measured accordingly. Single scalar measurements may also ignore the fact that smaller units are embedded in larger spatial contexts. Within larger entities with low or moderate segregation levels, strong concentrations may exist at smaller spatial scales, or vice versa. Focusing on only one spatial scale may overlook specific ethnic concentrations (Fowler 2016). Furthermore, fixed borders may lead to over- or underestimations of very specific concentrations that occur at the border of two administrative or statistical districts (Clark et al. 2015).

The increased availability of geo-coded individual data offers opportunities for solving boundary and scale issues by constructing scalable individualised neighbourhoods. These districts are ‘egocentric’: the exact residential location of an individual is taken as the centroid, from which point a buffer is constructed that consists of a predefined distance radius (Reardon et al. 2008) or a k-number of nearest neighbours (*k*-levels) (Andersson and Malmberg 2015; Östh et al. 2014). The resulting sample of individuals is then used to compute aggregate statistics, such as the share of people within a buffer belonging to a certain migrant group (Clark et al. 2015). Since the distance radius or the number of nearest neighbours within the buffer can vary, individualised neighbourhoods of different sizes seen from the same location can be studied, enabling the analysis of residential segregation from a multiscalar perspective.

Scale can be of importance both with respect to concentration and representation. If there is a high concentration of non-European migrants at small neighbourhood scales (for example, among the nearest 200 neighbours) but not on a larger scale (for example among the 1000 or 10,000 nearest neighbours), this can, as suggested by van der Wusten and Musterd (1998), have consequences for the way people living in the neighbourhood interact with different groups. Even if interactions with the closest neighbours will be mostly with non-European migrants, interactions in workplaces, at shopping centres, cafés, etc., can occur with a more mixed population. If, on the other hand, the concentration of non-European migrants is also high for larger-scale neighbourhoods including the 50,000 nearest neighbours, this can result in much larger proportions of individuals’ daily and weekly activities taking place in a context where the concentration of non-European migrants is high. In the literature, this has been related to mechanisms of role modelling and networks that may influence people’s norms and behaviours in these neighbourhoods. Swedish studies using multiscalar measures of neighbourhood contexts suggest that this reasoning is valid. Large-scale elite environments and large-scale deprived areas tend to have a more pronounced effect on individual-level outcomes compared to small-scale contexts (Andersson and Malmberg 2015, 2016).

A similar argument can be made with respect to the representation of non-European migrants. Certainly, high levels of small-scale, under- and over-representation of non-European migrants are problematic from a social cohesion perspective since they can signal a principle of separateness on the part of the native population and may also reflect a negative attitude towards mixing in the local area. Still, if the under- and over-representation of non-European migrants at higher scale levels is less strong, this can signal preparedness for sharing a broader urban environment with groups of different origins. Strong under- and over-representation at larger scales, for example, among the nearest 50,000 neighbours, could make under-represented groups feel unwelcome. Living in an area where large-scale under-representation is strong also implies that there is little opportunity, even by moving outside your immediate neighbourhood, of getting to know the under-represented group.

## Data and Methods

The empirical analysis of this paper focuses on non-European migrants, that is, foreign-born individuals with a country of birth outside Europe (defined as consisting of the 28 European Union member states plus the four EFTA countries, Norway, Switzerland, Iceland and Liechtenstein). It is important to realise that this definition implies that we study only first-generation immigrants and not their descendants. In all the countries, data on country of birth are from population registers (the central population register in Denmark, the national register of natural persons in Belgium, municipal population registers in the Netherlands, and the total population register in Sweden).

In Denmark, Belgium, and the Netherlands, residential coordinates for individuals in the population registers were obtained by matching addresses in the population registers with addresses in building or land registers (Den Offentlige Informationsserver [OIS] in Denmark, Land Registry of the General Administration of Patrimonial Documentation in Belgium, Basisregistratie Adressen en Gebouwen in the Netherlands). In Sweden, permanent residents are registered to specific real estate properties and residential coordinates were obtained by matching with the land registry on the basis of the property registration numbers. For all four countries, data from 2011 were used.

In Sweden, the individual geo-coded data were made available to researchers through an online database created for the Department of Human Geography by Statistics Sweden (Geostar). In the Netherlands, the data are part of the System of Social Statistical Datasets (SSD) created by Statistics Netherlands. In Belgium, the linkage between the population register and the geo-coordinates is administered as a part of the 2011 register-based census. In each country, the total population was taken into account (Nielsen et al. 2017).

The processing of the register data was carried out in the same way in all four countries. In the first step, the individual-level data were aggregated to a geographical grid of 100 by 100 m (in Denmark, the Netherlands, and Belgium) and, for Sweden, to a geographical grid of 250 by 250 m (in densely populated areas, due to data restrictions), or 1000 by 1000 m (in sparsely populated areas); see Table 1. For each square, the number of non-European migrants and the total population number were computed.

**Table 1:** The gridded population, descriptive statistics, 2011. *Source*: Authors’ calculations based on register data from statistics Belgium, statistics Denmark, statistics Netherlands, and statistics Sweden

	Number of populated grid squares	Median population	Maximum population	Median number of non-European migrants	Maximum number of non-European migrants	Total population
Belgium	608,850	9	1753	0	516	11,000,638
Denmark	421,365	5	1129	0	275	5,566,100
Netherlands	559,504	11	1105	0	771	16,727,659
Sweden	202,067	157	4114	7	1345	9,466,727

In the second step, Equipop software (Östh et al. 2014) was used to process this gridded population data. Equipop expands a buffer around each populated grid cell until the total population count in the buffer reaches a threshold level of *k* nearest neighbours. When this threshold is reached, Equipop computes the proportion of non-European migrants in the buffer population. If calculations for multiple k-levels are requested, the software then continues to expand the buffer until the next threshold is reached, computes the proportion of non-European migrants, and continues to expand the buffer and calculate proportions until values for all the requested k-values are obtained. In the current study, we focus proportions of non-European migrants computed for the 200, 1600, 12,800 and 51,200 nearest neighbours.

As the individualised neighbourhoods are expanded until a specific population threshold is reached, their geographical size/radius in metres will vary depending on population density. Table 2 provides information about this variation. Some neighbourhoods will be very large, much larger than the areas one conventionally regards as neighbourhoods. Thus, our concept of neighbourhood is stretched in its meaning. They are neighbourhoods by virtue of being areas that reach a predefined number of closest neighbours.

**Table 2:** Size of individualised neighbourhoods in Belgium, Denmark, the Netherlands, and Sweden, radius in metres (percentiles based on population count), 2011. *Source*: Authors’ calculations based on register data from statistics Belgium, statistics Denmark, statistics Netherlands and statistics Sweden

Percentile	Belgium	Denmark	Netherlands	Sweden
	*k* = 200	*k* *=* 200	*k* *=* 200	*k* *=* 200
10	100	100	100	0
25	100	100	100	0
50	141	141	100	250
75	224	224	141	250
90	424	1000	224	1414
95	608	1513	500	2236
99	1105	2200	1265	5000

	*k* *=* 51,200	*k* *=* 51,200	*k* *=* 51,200	*k* *=* 51,200
10	1500	1664	1712	2000
25	2865	3354	2302	3162
50	5049	7912	3612	10,050
75	7200	15,008	6379	22,472
90	9411	20,132	9080	35,609
95	12,394	23,308	10,515	44,294
99	20,096	36,111	14,091	104,346

One important conclusion from Table 2 is that in spite of large differences in overall population density, people in the four countries live in local neighbourhoods that are similarly structured. Fifty per cent of the population have their closest 200 neighbours within approximately 200 m from their dwelling or less, and 90% of the population have their closest 200 neighbours within approximately 1000–1500 m or less, and in Belgium, as close as within a 400-m radius. Only approximately 1% of the population in Sweden lives in a location where the distance to the closest 200 neighbours is much greater than in Denmark, Belgium, or the Netherlands.

If, however, the neighbourhood scale is expanded to encompass the closest 51,200 neighbours, the picture changes somewhat. For 25% of the population, there is essentially no difference. Their distance to the nearest 51,200 neighbours is approximately 3 km or less in all four countries. Instead, the largest differences in population density are found for the 25% of the population that live in the sparsely populated areas. In Sweden, for this population, the neighbourhood area must be given a radius of at least 22.5 km in order to encompass 51,200 neighbours, whereas 11 km or more will suffice to reach 51,200 for the Dutch and Belgians living in the 25% of the most sparsely populated neighbourhoods. Furthermore, the differences are even larger for the 1% most sparsely populated individualised neighbourhoods. In Sweden, this group would need to attract all neighbours within a radius of at least 100 km in order to reach 51,200 people, whereas in the Netherlands, a radius of 16 km would suffice. Thus, in spite of large differences in population density, national differences in the geographical structure of the neighbourhoods are unlikely to influence segregation patterns at small neighbourhood scales. On the other hand, it could be argued that in sparsely populated parts of Sweden, the presence of non-European migrants among the closest 51,200 neighbours implies that there is not even cycling distance between them and individuals from the majority population.

### Concentration

The values obtained as output from the Equipop processing correspond to the concentration measure of segregation: the proportion of the local neighbourhood’s population that consists of non-European migrants. In order to compare differences in concentration across countries, we look at the percentile values of this proportion. One important point to remember here is that when neighbourhood values are computed for individualised neighbourhoods, the number of neighbourhoods will correspond to the number of individuals in the population. Each individual has its own neighbourhood. Thus, if the 10^th^ percentile of the local neighbourhoods’ population that consists of non-European migrants is 1%, this signifies that 10% of the population lives in neighbourhoods with less than 1% non-European migrants. Percentile values across different countries and across *k*-values will therefore provide a detailed and comparable picture of how segregation in terms of the local concentration of non-European migrants varies between countries.

### Representation of Non-European Migrants

Representation is measured as the proportion of the total non-European migrant population that lives in a neighbourhood. If this proportion is lower than that neighbourhood’s proportion of the overall total population, then non-European migrants are under-represented. To assess this representation of non-European migrants in different neighbourhoods, the data resulting from the Equipop processing were aggregated into 100 different neighbourhood types, or bins, based on the proportion of non-European migrants, with each bin representing 1% of the total population. For details, see Andersson et al. (2017).

Based on these grouped neighbourhoods, the representation of non-European migrants in different types of neighbourhoods was computed as:

1 $$\frac{{Non\;Europeans_{i} }}{{\mathop \sum \nolimits_{i = 0}^{99} Non\;Europeans_{i} }}$$

where *Non Europeans*_*i*_ is the number of non-European migrants living in bin *i*. Expression (1) is thus the proportion of the total non-European migrant population, ∑ _*i*=0_^99^*Non Europeans*_*i*_, that is living in bin *i*. As each bin contains 1% of the total population, equal representation is achieved when the value of expression (1) is 1%. If the proportion is lower than 1%, non-Europeans are under-represented. If the proportion is higher than 1%, non-Europeans are over-represented.

The definitions of concentration and representation used in this paper are given in Table 3. Here, it is clarified that the concentration measure relates the size of any given neighbourhood’s non-European migrant population to the size of the total neighbourhood population, whereas the representation measure relates the size of any given neighbourhood’s non-European migrant population to the total non-European migrant population in the country (Hennerdal and Nielsen 2017). Note also the different probability interpretations of concentration and representation that are given in Table 3.

**Table 3:** Concentration and representation explained

Concentration	Representation
$\frac{Non\;European\;migrants\; in\;neighborhood}{Total\;neighborhood\;population}$	$\frac{Non\;European\;migrants\;in\;neighborhood}{Total\;Non\;European\;migrant\;population}$
*Probability interpretation*: Selecting one individual randomly from the neighbourhood, what is the probability that the individual will be a non-European migrant?	*Probability interpretation*: Selecting one individual randomly from the non-European migrant population, what is the probability that the individual will live in this specific neighbourhood?

### The Dissimilarity Index: An Aggregate Measure of Over- and Under-Representation

The most widely used aggregate measure of segregation is the dissimilarity index (Duncan and Duncan 1955; Massey and Denton 1988). With two population groups, *NE*, non-European migrants, and *E,* European-born persons, the dissimilarity index can be defined as:

2 $$DI = \frac{1}{2}*\mathop \sum \limits_{i = 0}^{99} \left| {\frac{{ne_{i} }}{NE} - \frac{{e_{i} }}{E}} \right|$$

where *ne*_*i*_ is the number of non-European migrants living in neighbourhood bin *i*, *e*_*i*_ is the number of European-born persons living in neighbourhood bin *i, NE* is the total non-European migrant population, and *E* is the total European-born population. An inspection of (2) shows that $\frac{{ne_{i} }}{NE}$ is our measure of representation for non-European migrants. In the same way, $\frac{{e_{i} }}{E}$ is the representation of European-born persons. This implies that the dissimilarity index, being the sum of the absolute difference between non-European migrant representation and European-born person representation divided by two is an aggregate measure of over- and under-representation. *DI* will be zero if both groups are equally represented in all neighbourhoods, and one if non-European migrants have zero representation in neighbourhoods where European-born persons live, whereas European-born persons have zero representation where non-European migrants live. Note that in this formula, *NE, ne*_*i*_, *E, and e*_*i*_ are not defined in the same way as when *DI* is computed for fixed geographical areas. Yet, the standard interpretation of *DI* as the share of the minority population that must move in order to arrive at an even distribution still applies.

Using the approach described above, we computed dissimilarity indices for the four countries under study and for different k-values. A comparison of these indices will complement the analysis of over under- and over-representation based on percentile plots and help us to assess the extent to which segregation patterns in these countries are similar or dissimilar.

## Results

The first point of interest is how the inflow of non-European migrants has shaped the population *composition* of neighbourhoods in the four countries under study. This concentration can be analysed using bin plots for the proportion of the neighbourhood population that consists of non-European migrants. Another point of interest is the extent to which the non-European migrant population is evenly distributed across neighbourhoods. In other words, the analysis focuses on the degree to which non-European migrants are *over*- and *under*-*represented* in neighbourhoods.

### The Non-European Migrant Population in Belgium, Denmark, the Netherlands, and Sweden

There are few studies that have systematically studied the extent to which segregation levels differ between countries. Therefore, the aim of this study is to compare levels and patterns of non-European migrant segregation in the four different countries.

Non-European migrants^1^ in the four countries under study are more similar in terms of their origins compared to European migrants, whose origins are more diverse and distance-related. If there is a crisis, e.g., in a Middle Eastern country, immigrants will enter all countries examined in this study. Differences between the European migrant populations also occur because European immigrants are dominated by the closest neighbouring countries, like Finns and Norwegians in Sweden, for example. These immigrants often dominate in border regions.

The total population of Denmark in 1990 was 5,135,409, and in 2011, it was 5,560,628 and the proportion of immigrants increased over the same period. Among all immigrants, the proportion of non-EU immigrants has risen the fastest, from 1.9 in 1990 to 4.8 in 2011 (and 5.7 in 2016).

The total Swedish population increased from 8,590,701 in 1990 to 9,555,892 in 2012. Compared to Denmark, Sweden had a higher proportion of both European and non-European migrants at the beginning and end of the period. An important difference is that in Sweden during this period, the proportion of European migrants was higher compared to Denmark, where non-European migrants represented the majority of migrants.

In the Netherlands, the composition of migrants is similar to Denmark in that the proportion of non-European migrants is larger than the proportion of European migrants. The four largest non-Western groups in the Netherlands are Moroccans, Antilleans, Surinamese and Turks (Hartog and Zorlu 2009).

In Belgium, Europeans make up the majority of migrants, coming mostly from neighbouring countries as well as from Italy. Nevertheless, the largest increase in numbers in recent years was Eastern Europeans after the successive enlargements of the EU since the 1990s. The largest groups of non-European migrants in Belgium are of Turkish, Moroccan, and to a lesser extent, Congolese origin (Phalet et al. 2007).

### Neighbourhood-Level Concentration of Non-European Population: By Percentiles

**Fig. 1:**
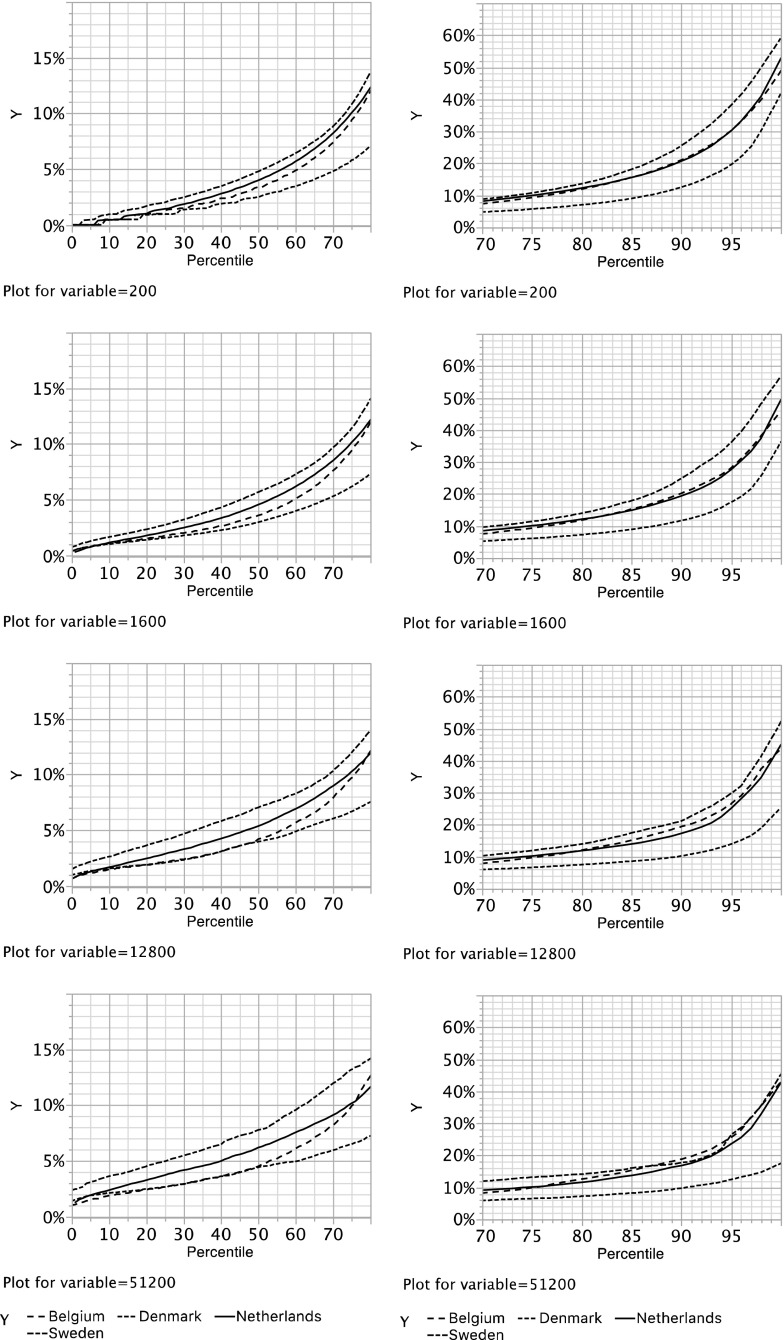
Concentration of non-European migrants in individualised neighbourhoods in Belgium, Denmark, the Netherlands and Sweden, 2011. Percentile values for *k*-levels 200, 1600, 12,800 and 51,200. Lower percentiles in column one and percentiles above 70 in column two

In Fig. 1, percentile plots for different scales (*k*-levels) show how the concentration of non-European migrants varies between neighbourhoods across countries. The percentile plots are split into two parts: one plot showing percentiles 0–80 (presented in the left-hand column of Fig. 1) and one plot showing percentiles 70–99 (presented in the right-hand column). By splitting the plots, *different scaling of the vertical axes* can be used, making it easier to read the concentration values for low percentiles. In the discussion below, we first review percentiles with the highest proportions of non-European migrants, especially the 10% most immigrant-dense areas. Thereafter, we look at percentiles consisting of neighbourhoods with low proportions of non-European migrants.

Concerning the rank of the proportions in the various countries, see the right column, first diagram in Fig. 1, for *k* = 200 and the 90–99% of the population living in the most non-European-dense areas. The line for Sweden is above the others, showing the highest concentrations of immigrants in these immigrant-dense neighbourhoods. The reason for this is based on the fact that Sweden has the highest proportion of non-European migrants overall, while the Netherlands, followed by Belgium and then Denmark, have lower levels (Table 4). The most important message, however, is that the patterns of the proportions across neighbourhoods of non-European migrants are very similar across countries; it is almost exclusively the difference between the overall proportions that makes the difference between lines in the graphs.

**Table 4:** Population share of non-European migrants in Belgium, Denmark, the Netherlands and Sweden, 2011, per cent. *Source*: Authors data and Eurostat

Country	2011 (%)	2015 (Jan., 1) born in non-member state (Eurostat) (%)
Denmark	4.8	6.9
Belgium	7.3	8.5
Netherlands	8.0	8.7
Sweden	9.1	11.1

In Denmark, in the 10% most migrant-dense areas, proportions vary from 13% up to the value of 36% (at the *k* = 200 level) for the 90th to 99th percentiles (Table 5). These proportions are lower compared to the most immigrant-dense areas in the other countries. In Belgium, the proportions are a little higher: 21–44% non-European migrants in the densest areas. For the Netherlands, the proportions are higher again, with equivalent numbers of 21–47% non-European migrants in the 10% most immigrant-dense neighbourhoods. In Sweden, the percentage for 2011 is the highest and varies between 26 and 55% non-European migrants in the most immigrant-dense areas; see Table 5 and Fig. 1. Thus, concentrations of non-European migrants in the Netherlands and Sweden are high in certain neighbourhoods.

**Table 5:** Concentration of non-European immigrants in Belgium, Denmark, Netherlands and Sweden, percentiles for different scales (*k*-levels), 2011. *Source*: Authors’ calculations based on register data from statistics Belgium, statistics Denmark, statistics Netherlands and statistics Sweden

Percentile	Belgium	Denmark	Netherlands	Sweden
	*k *= 200	*k *= 200	*k *= 200	*k *= 200
10	0.5%	0.5%	0.5%	0.9%
25	1.3%	1.0%	1.4%	2.0%
50	3.4%	2.5%	4.0%	4.8%
75	9.4%	5.8%	10.0%	10.8%
90	21.1%	12.6%	20.7%	25.5%
95	30.3%	19.6%	30.2%	38.3%
99	44.4%	36.1%	46.8%	54.6%

	*k *= 1600	*k *= 1600	*k *= 1600	*k *= 1600
10	1.1%	1.0%	1.2%	1.7%
25	1.8%	1.6%	2.1%	2.8%
50	3.6%	3.0%	4.6%	5.7%
75	9.4%	6.2%	10.1%	11.4%
90	20.3%	11.7%	19.5%	24.9%
95	28.2%	17.5%	27.7%	36.2%
99	42.1%	31.0%	43.4%	52.6%

	*k *= 12,800	*k *= 12,800	*k *= 12,800	*k *= 12,800
10	1.5%	1.6%	1.7%	2.6%
25	2.2%	2.1%	2.9%	4.1%
50	4.2%	4.0%	5.4%	7.1%
75	9.7%	6.7%	10.2%	12.0%
90	19.4%	10.3%	17.3%	21.2%
95	26.6%	13.9%	25.1%	29.9%
99	40.6%	22.2%	39.8%	46.7%

	*k *= 51,200	*k *= 51,200	*k *= 51,200	*k *= 51,200
10	1.9%	2.1%	2.4%	3.6%
25	2.7%	2.7%	3.7%	5.0%
50	4.5%	4.4%	6.2%	7.8%
75	9.9%	6.5%	10.1%	13.2%
90	18.8%	9.8%	16.8%	17.7%
95	25.6%	12.5%	23.6%	26.5%
99	39.3%	16.1%	37.8%	40.4%

The left-hand column in Fig. 1 shows the neighbourhoods with low proportions of non-European migrants, which is where natives and European migrants dominate. Continuing from the discussion above, the overall rank of countries’ proportions in neighbourhoods shows only a few deviations from this pattern, reflecting the national proportions. There are also two groups of countries, one including Sweden and the Netherlands and another including Denmark and Belgium. Seventy per cent of the population (percentiles > 30) in Sweden live in diversifying neighbourhoods, that is, neighbourhoods containing more than 5% non-European migrants (*k* = 12,800, in Fig. 1). The situation in Sweden can be considered as one in which there is a move towards spatial assimilation with regard to having diverse neighbourhoods (even if this is only a cross-sectional measurement). Non-European migrants are living in the majority of neighbourhoods.

Belgium, on the other hand, shows a pattern of low proportions in neighbourhoods of approximately 50% of its population, but then there is an abrupt change to high proportions of non-European migrants in the remaining 50% of the population (*k* = 12,800 in Fig. 1). Hence, even if Belgium and Denmark have similar low levels of non-European migrants in low migrant-dense areas, the lines depart from 50% of neighbourhoods. This is especially clear at higher scale levels of *k* = 12,800 and 51,200. In Belgium, the pattern indicates that large areas of the housing stock are inaccessible to non-European migrants. This may be due to the fact that many non-European newcomers are concentrated in central urban neighbourhoods with low-quality dwellings and are excluded from suburban areas with better housing conditions. The analogous line for Belgium is at the same low proportion level as for Denmark, which has a much lower overall proportion of non-European migrants. Nevertheless, the line for Denmark in Fig. 1 continues at a lower level of non-European migrants in neighbourhoods for the entire population.

### Representation of Non-European Migrants Across Neighbourhoods

**Fig. 2:**
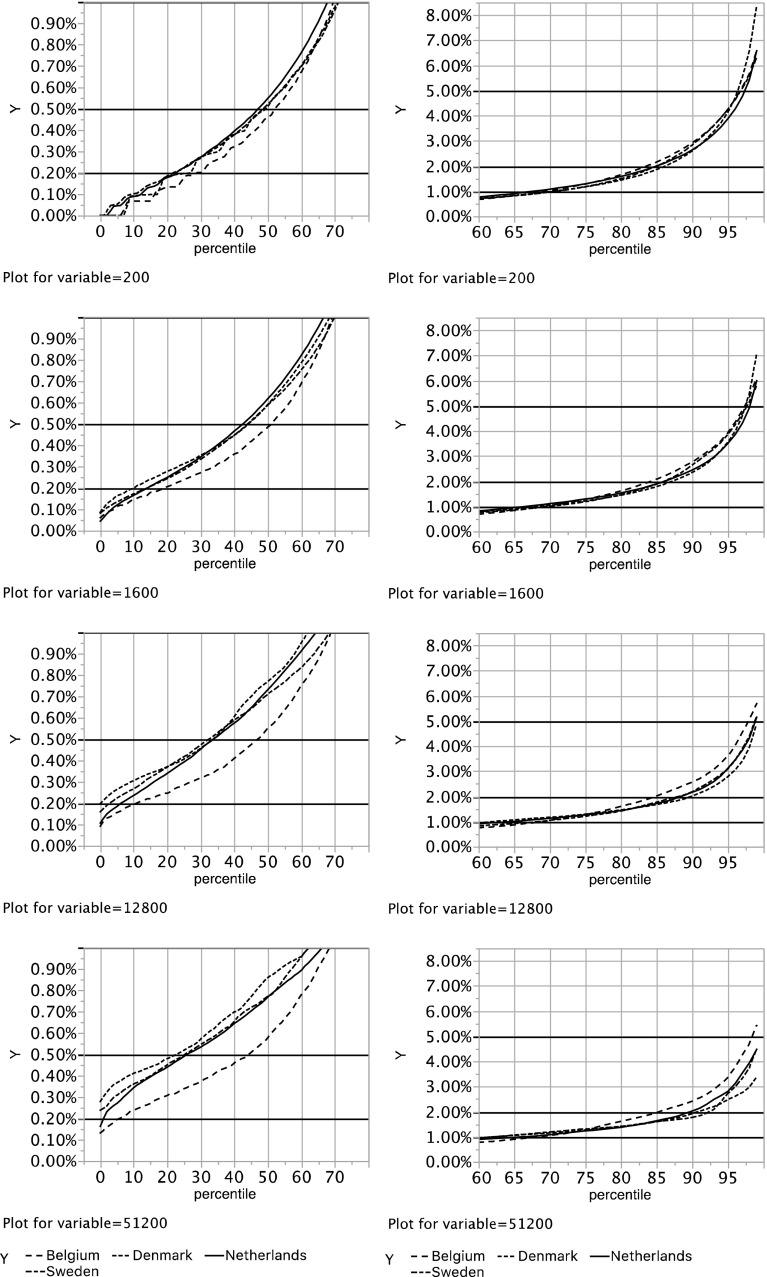
Representation of non-European migrants in 1% population bins, 2011. Population bins sorted according to proportion of non-European migrants and diagrams showing different *k*-values. Left column showing under-representation (below 1%, which is at the top of the diagram) and moderate and strong under-representation with 0.5 and 0.2%. Right column illustrating over-representation above 1% and moderate and strong over-representation at 2.0 and 5.0% non-European migrants in a bin. See online appendix for a discussion of these values

Figure 2 shows, for different *k*-values, the *representation* of non-European migrants across neighbourhood types in Belgium, Denmark, the Netherlands, and Sweden. Again, in order to facilitate the analysis, the left-hand column shows this proportion for the 71 bins that have the lowest proportions of non-European migrants. The right-hand column shows the proportion of non-European migrants living in the 41 bins with the highest proportion of non-European migrants. Remember also that each bin represents 1% of the total population in each country; that is why there should be 1% of non-European immigrants if they were evenly distributed across neighbourhoods.

First examining the left-hand column Fig. 2, these graphs show how large parts of the population in the four countries live in neighbourhoods with *a lower proportion* of non-European migrants than one would expect if every bin had the same proportion of non-European migrants (top of graph is equal to 1%).

If the proportion of the total population of non-European migrants in the different bins had been close to 1%, this would indicate low levels of segregation. In contrast, the graphs show that large parts of the population in all these four countries live in neighbourhoods where the proportion of non-European migrants is much lower than would be expected if there was no segregation. This is especially true for low *k*-levels. For *k* = 200, approximately 50% of the population in Belgium, Denmark, the Netherlands and Sweden live in neighbourhoods whose proportion of non-Europeans is less than half (0.5%) of what would be expected with an equal distribution of non-Europeans across neighbourhoods. Along these lines, 20% of the population in all Denmark, the Netherlands and Sweden, and close to 30% of the population in Belgium live in neighbourhoods whose proportion of non-European migrants is less than one-fifth of what would be expected with an equal distribution. What is striking here is that the figures are very similar across Denmark, the Netherlands and Sweden, Fig. 2. Belgium has an even stronger under-representation.

For larger *k*-values, segregation levels are less strong, at least in Denmark, the Netherlands and Sweden. Thus, for *k* = 51,200, less than 25% of the population in Denmark, the Netherlands and Sweden live in neighbourhoods whose proportion of non-European migrants is less than half of what would be expected with an equal distribution. Furthermore, almost no one lives in areas where the proportion of non-Europeans is less than one-fifth of what would be expected with an equal distribution. Again, Belgium differs. Here, almost 45% of the population lives in neighbourhoods whose proportion of non-Europeans is less than half of what would be expected with an equal distribution. Moreover, approximately 5% of the Belgian population lives in neighbourhoods where the representation of non-European migrants is less than one-fifth of what would be expected with an equal distribution.

Turning to the right-hand column, we focus instead on segregation patterns in areas where there is over-representation of non-European migrants. As shown in these graphs, this over-representation starts close to the 70th percentile for low k-values and at around the 60th percentile for the highest k-values. For *k* = 200, however, only approximately 15% of the population in Denmark, the Netherlands and Sweden (and somewhat more in Belgium) lives in neighbourhoods whose proportion of the total non-European population is twice as high as it would be with no segregation. For higher k-values, this percentage is even lower, at least if Belgium is excluded. In Sweden, for *k* = 51,200, about 7.5% of the population lives in neighbourhoods where the representation of non-Europeans is twice the level of equal representation. In Denmark and the Netherlands, 10% of the population lives in such neighbourhoods, whereas in Belgium, approximately 15% of the population lives in areas with such over-representation. Moreover, in Belgium, 2% of the population lives in *k* = 51,200 neighbourhoods where the over-representation of non-European migrants is even more extreme, 7.5 times the level that would correspond to equal representation.

### Dissimilarity Index

The third indicator we use in our study is the dissimilarity index (DI) showing the extent to which the spatial sorting of the non-European migrant population is stronger in some of our studied countries than in others. The smaller the number of nearest neighbours, the larger the measured dissimilarity index. This is the regular consequence of small populations becoming homogenous more easily than large populations and areas. The larger the population, the more likely there will be a larger mix and a lower dissimilarity index. This is undoubtedly the most obvious result when measuring dissimilarity at several scales, and points to the importance of having detailed multiscalar data when measuring segregation, as shown in Table 6.

**Table 6:** Dissimilarity index in Belgium, Denmark, Netherlands and Sweden, 2011. *Source*: Authors’ calculations based on register data from statistics Belgium, statistics Denmark, statistics Netherlands and statistics Sweden

*k*-value	Belgium (%)	Denmark (%)	Netherlands (%)	Sweden (%)
200	51.2	47.5	48.7	48.9
1600	47.3	40.4	43.6	44.1
12,800	43.7	31.3	37.5	35.7
51,200	40.6	25.3	32.6	29.7

Starting with the 200 closest neighbours, i.e., rather small local areas, the dissimilarity index ranges from Denmark’s 0.475 to the value for the Netherlands of 0.487, to Sweden’s value of 0.489 and the highest dissimilarity for Belgium of 0.512. The same order, with Denmark having the lowest, the Netherlands second lowest, Sweden third lowest and Belgium the highest measured DI is the rule throughout *k* = 200, 400, 800, 1600, 3200 and 6400. At the large-scale level of neighbourhoods with 12,800 closest neighbours, the order is changed so that the Netherlands has the second highest value followed by Sweden and Denmark, and the same goes for *k* = 51,200. In all cases, Belgium has the highest value for DI. While Sweden and the Netherlands by scale change from the second to third highest index value, Denmark increases the relative difference in DI value compared to Sweden and the Netherlands as the scale of neighbourhoods increases. In conclusion, Denmark has the lowest segregation of non-European migrants at all scale levels as measured by the dissimilarity index.

## Discussion and Conclusions

The aim of this paper has been to compare levels and patterns of non-European migrant segregation in four different countries. We have analysed the extent to which there are differences in the concentration and representation of non-EU migrants across various spatial scales.

Our first finding is that the differences we find between countries are small, and moreover, that the similarities are especially pronounced at the lowest scale level. This is in sharp contrast to earlier studies that reported large differences in the dissimilarity index between urban areas, e.g., Skifter Andersen et al. (2016), Musterd (2005) and Arbaci (2007). One reason for this could be that we focus on entire national areas, but it can also reflect that we have tried to measure segregation for comparable migrant groups in a way that avoids the MAUP. Again, our contribution is that the results we present show that use methods ensuring comparability and multiscalability to provide possibilities for evaluating competing theories of segregation and assessing the impact of different policies.

If one focuses on larger neighbourhood scales, however, we do find differences. Here, as is clearly evidenced by the dissimilarity index, Belgium stands out as the exception, whereas Sweden, Denmark, and the Netherlands are remarkably similar. The fact that the similarities are stronger for small-scale than for large-scale patterns underlines the importance of using a multiscalar approach to segregation measurement.

A possible explanation for the similarities in large-scale segregation patterns between Sweden, Denmark, and the Netherlands, as well as the contrast with Belgium, is how the housing sectors in these countries have developed since the 1950s. For an extended period, Sweden, Denmark, and the Netherlands put strong emphasis on the construction of relatively large housing estates that have provided relatively low cost and accessible housing options. With increasing incomes, these housing options often became a secondary option for middle-income households preferring single-family housing, with the result that these dwellings have become an important alternative for newly arrived migrants with relatively low incomes. At the same time, the public housing sector in Sweden, Denmark, and the Netherlands is not restricted only to the poorest groups, and this may have stimulated some mixing. Social housing in Belgium, in contrast, is typically more exclusively for the poor and this may have contributed to a higher degree of segregation. Another difference is that housing allowances are much more widely available in Sweden, Denmark, and the Netherlands compared to Belgium (Juntto and Reijo 2010). As housing allowances also make housing options in less low-income dominated areas more available for low-income households, this can make it possible for poor, non-European migrant households to access neighbourhoods preferred by medium-income European-born households.

However, it could be that differences in immigrant settlement policies have also played a role. Such policies have often been deemed to have little effect but a more detailed exploration of whether policies of dispersal have affected Denmark, Sweden, and the Netherlands is required to prevent the higher segregation levels at larger scales that characterise Belgium.

In terms of concentration (Fig. 1), the differences between the four countries studied here are much larger than differences in the patterns of representation. This is because the relative size of the non-European migrant population differs between the four countries. Thus, Sweden, having the highest proportion of non-European migrants in its population, also has the highest proportion of the population living in migrant-dense neighbourhoods, at least for small- to medium-sized neighbourhoods. Denmark, having the lowest proportion of non-European migrants in its population, also has the lowest proportion of the population living in migrant-dense neighbourhoods. The conclusion here is that by increasing the levels of concentration of migrants in migrant-dense neighbourhoods, an expanding migrant population may accentuate problems associated with segregation even in cases where there is no change in patterns of over- and under-representation.

Analyses of segregation among foreign-born migrants should focus not only on migrant-dense neighbourhoods, however, but also on areas where migrants are under-represented. In many such areas, both Sweden and the Netherlands have relatively high concentrations of non-European migrants.

In Sweden, especially for larger-scale levels, only very small parts of the population live in neighbourhoods with low concentrations of non-European migrants. For example, for *k* = 51,200, less than 20% of the population lives in neighbourhoods with fewer than 5%. In Denmark and Belgium, more than 50% of the population lives in such native- and European migrant-dominated neighbourhoods. Although spatial assimilation is a process that evolves over time, this pattern in Sweden could be interpreted as a sign of the start of spatial assimilation. The stronger persistence of neighbourhoods with very low concentrations of non-European migrants in Denmark and Belgium can be interpreted as a reflection of place stratification, namely that there are areas where non-European migrants are more or less excluded from entry. That both Denmark and Belgium have a lower proportion of non-European migrants certainly contributes to this pattern, but it could also be the case that an expanding migrant population is an important factor affecting spatial assimilation. The expansion of a migrant population leads to a spill-over effect when early and established migrants settle in areas with lower concentrations. Here, it should be noted, however, that Belgium has a higher proportion of non-European migrants in the population than Denmark. The fact Belgium has the same high proportions of the population in neighbourhoods with few non-European migrants is therefore the result of stronger spatial sorting in Belgium. One interpretation of this pattern is that place stratification is stronger in Belgium than in Denmark.

Many studies have shown that non-European migrants, especially the newly arrived, have higher unemployment and lower income levels than the general population (Semyonov and Gorodzeisky 2014). Because of this disadvantage, the high concentrations of non-European migrants found in the highest percentiles in the countries under study indicate a risk of negative neighbourhood effects. In response to this, several countries have developed dispersal policies, although such policies can be seen as problematic with regard to the individual’s right to decide settlement. These policies can also be problematic if they assign individuals to areas with few employment opportunities. In the case of Denmark, an infamous ‘ghetto’ debate is the result of such initial thoughts about high concentrations (Aner 2015). In contrast, the present study shows no such evidence of Denmark having especially high levels of segregation compared to the other studied countries.

Given that neighbourhood effects are probably stronger when there is strong segregation across scale levels, living in large housing estates built during the 1960s and 1970s might affect residents more. Large-scale segregation has the consequence of producing environments that meet most of their residents’ needs. As mentioned as early as 1998 in the book edited by Musterd, a self-sufficient environment with larger units does not provide any incentive to use the wider urban space. On the other hand, small-scale segregation, where there is a high proportion of non-European migrants among the 200 closest neighbours, might be small and difficult to interpret in terms of neighbourhood effects if the nearest neighbourhood has a diverse population composition.

In the case of Belgium, where the dissimilarity index was particularly high, social cohesion might be a concern. Nationally, Belgium shows low levels of non-European migrants in many neighbourhoods, but the gap in the size of the concentration is high in that there are also neighbourhoods with very high proportions of non-European migrant residents. Compared to the other three countries, larger-scale segregation of non-European migrants is also much stronger in Belgium. Such pronounced residential segregation patterns provide fewer opportunities for diversity and mixing in neighbourhoods, which in the long run might hinder understanding between groups and social cohesion in society.

## Conclusions

An important first conclusion of this paper is that small-scale segregation patterns of non-European migrants are strikingly similar across the four countries studied in this paper. To our knowledge, such a consistency has not been demonstrated in any earlier studies and it provides a challenge to future attempts to explain processes of segregation. A second conclusion is that there is more variation between countries with respect to large-scale segregation patterns. This indicates that factors explaining large-scale segregation can be different from factors that explain small-scale segregation. We have not been able to explicitly test different explanations. Yet, in line with our summary of earlier studies, we find that differences in housing policies can certainly be important. Finally, even if all four countries under study have neighbourhoods with high concentrations of non-European migrants, it is also the case that in Denmark, the Netherlands and Sweden, at least, substantial numbers of non-European migrants can also be found outside such migrant-dense areas. The popular image that non-European migrants are concentrated only in migrant-dense areas is not consistent with the results of this paper.

## Electronic supplementary material

Below is the link to the electronic supplementary material.

Supplementary material 1 (DOCX 12 kb)

## Footnotes

1 We subsequently use the definition EU28+EFTA. This does not include all European countries. In the text, we consider people from Andorra or Serbia as non-Europeans.
