# Supplementary material for: A Comparative Study of Segregation Patterns in Belgium, Denmark, the Netherlands and Sweden: Neighbourhood Concentration and Representation of Non-European Migrants
Source: Eur J Popul. 2018 Mar 21;34(2):251–75. doi: 10.1007/s10680-018-9481-5 (PMC5932096; doi:10.1007/s10680-018-9481-5)
Supplement: Supplementary file 1 — Supplementary material 1 (DOCX 12 kb) [file 10680_2018_9481_MOESM1_ESM.docx]

# On-line appendix

A comparative study of segregation patterns in Belgium, Denmark, the Netherlands and Sweden:

Neighbourhood Concentration and Representation of Non-European migrants

## Break-point values for over- and under-representation

Patterns of over- and under-representation of non-European migrants were analysed using plots that showed the variation in representation across neighbourhood bins. Two sets of plots were produced; one set focusing on neighbourhood bins where non-European migrants are under-represented, and one set of plots focusing on neighbourhood bins where non-European migrants are over-represented, that is, where the representation of non-European migrants is above one percent. Moreover, we suggest, due to reasons of interpretation, that representation values above 2 % and below 0.5 % can be designated as cases of *moderate* over- or under-representation respectively, and that representation values above 5 % and below 0.2 % can be designated as cases of *strong* over- and under-representation. Interpreting the numbers, one can say that 0.5% representation is half of what would be equal representation and 2%, twice the size of the equal representation value. Similarly, 0.2% is one-fifth of the norm, and 5% is five times the equal representation norm. Given the distribution of representation values in the four countries under study these cut-off values, included in the graphs (Figure 2), provide good benchmarks.
